# Light-driven increase in carbon yield is linked to maintenance in the proteorhodopsin-containing *Photobacterium angustum* S14

**DOI:** 10.3389/fmicb.2015.00688

**Published:** 2015-07-10

**Authors:** Alicia Courties, Thomas Riedel, Alain Rapaport, Philippe Lebaron, Marcelino T. Suzuki

**Affiliations:** ^1^Sorbonne Universités, UPMC Univ Paris 06, CNRS, Laboratoire d’Océanographie Microbienne (LOMIC), Observatoire Océanologique, Banyuls-sur-Mer, France; ^2^Sorbonne Universités, UPMC Univ Paris 06, CNRS, Laboratoire de Biodiversité et Biotechnologies Microbiennes (LBBM), Observatoire Océanologique, Banyuls-sur-Mer, France; ^3^INRA-Supagro, UMR MISTEA, Montpellier, France; ^4^INRA-INRIA, MODEMIC Team, Sophia Antipolis, France

**Keywords:** carbon yield, *Photobacterium angustum*, photoheterotrophy, Pirt model, proteorhodopsin

## Abstract

A type of photoheterotrophic bacteria contain a transmembrane light-driven proton pump called proteorhodopsins (PRs). Due to the prevalence of these organisms in the upper water column of the World’s Ocean, and their potential for light-driven ATP generation, they have been suggested to significantly influence energy and matter flows in the biosphere. To date, evidence for the significance of the light-driven metabolism of PR-containing prokaryotes has been obtained by comparing growth in batch culture, under light versus dark conditions, and it appears that responses to light are linked to unfavorable conditions, which so far have not been well parameterized. We studied light responses to carbon yields of the PR-containing *Photobacterium angustum* S14 using continuous culture conditions and light–dark cycles. We observed significant effects of light–dark cycles compared to dark controls, as well as significant differences between samples after 12 h illumination versus 12 h darkness. However, these effects were only observed under higher cell counts and lower pH associated with higher substrate concentrations. Under these substrate levels Pirt’s maintenance coefficient was higher when compared to lower substrate dark controls, and decreased under light–dark cycles. It appears that light responses by *P. angustum* S14 are induced by the energetic status of the cells rather than by low substrate concentrations.

## Introduction

A type of photoheterotrophic bacteria contain a transmembrane light-driven proton pump called proteorhodopsins (PRs). These organisms were discovered over a decade ago when a metagenomic fragment belonging to the gammaproteobacterial SAR86-cluster was shown to contain a gene with homology to the bacteriorhodopsins found in *Archaea* (Beja et al., 2000). PR activity generates a proton motive force (*pmf*) across the cell membrane, which has been postulated to lead to ATP generation by PR-containing prokaryotes (Beja et al., 2000). Although PR-based ATP generation has not been directly measured in the environment, PRs have been shown to be widely distributed (e.g., Beja et al., 2001; de la Torre et al., 2003; Rusch et al., 2007). More significantly a number of studies mostly using real-time PCR or genomic data (as PRs are difficult to detect directly) has shown that PR-containing prokaryotes might represent a large proportion of the total bacterioplankton of the upper water column (e.g., Rusch et al., 2007; Campbell et al., 2008; Riedel et al., 2010; Finkel et al., 2013). These high abundances, combined with the potential roles of PR-based ATP generation suggest that these organisms might have a significant impact on the ecology and biogeochemistry of marine systems. PR-based ATP generation has been hypothesized to have possible effects in growth rates (Gomez-Consarnau et al., 2007; Fuhrman et al., 2008), carbon metabolism (Fuhrman et al., 2008; Gonzalez et al., 2008), transport (Fuhrman et al., 2008), as well as starvation survival (Gomez-Consarnau et al., 2010; Steindler et al., 2011; Akram et al., 2013) among others.

Proteorhodopsin-coding genes are present in a wide taxonomic spectrum of microorganisms, particularly among environmentally abundant planktonic prokaryotes such as the alphaproteobacterial clades SAR11 (*Candidatus* Pelagibacter ubique; Giovannoni et al., 2005) and SAR116 (*Candidatus* Puniceispirillum marinus; Oh et al., 2010), the gammaproteobacterial clades SAR86 and SAR92 (Beja et al., 2000; Sabehi et al., 2004; Stingl et al., 2007a), the marine actinobacterial clade OM1 (*Candidatus* Actinomarinidae; Ghai et al., 2013), and the marine Euryarchaeota (Frigaard et al., 2006). PR-coding genes were also found in a number of strains belonging to groups frequently isolated from seawater such as the families Vibrionaceae (Gomez-Consarnau et al., 2010; Wang et al., 2012; Akram et al., 2013) and Flavobacteriaceae (e.g., Gomez-Consarnau et al., 2007; Gonzalez et al., 2008; Riedel et al., 2010).

Despite this relatively large amount of knowledge acquired for a group of bacteria discovered just over a decade ago, it is also remarkable that the significance of PR-based phototrophy in the ecology and biogeochemistry of the oceans remains poorly understood. Of primary significance, hypothesized effects of PR to global biogeochemistry, such as light stimulation of growth and carbon yield remain controversial, since experiments with different strains are non-concordant. So far, light stimulation of growth and yields under favorable conditions has only been shown for the flavobacterial strain *Dokdonia* sp. MED134 (Gomez-Consarnau et al., 2010; Gonzalez et al., 2011; Kimura et al., 2011), where stimulation has been attributed to changes in central metabolism (Palovaara et al., 2014). However, this strain appears to represent the exception, since in all other strains tested so far, either light stimulation on growth was not observed [e.g., SAR11 (Giovannoni et al., 2005), SAR92 (Stingl et al., 2007b), *Polaribacter* sp. MED152 (Gonzalez et al., 2008), and *Dokdonia* sp. PRO95 (Riedel et al., 2010, 2013)], or effects of light exposure were only observed under unfavorable conditions, such as an enhanced survival after prolonged nutrient limitation in *Vibrio* sp. AND4 (Gomez-Consarnau et al., 2010), respiratory stress in *Vibrio campbellii* BAA-1116 (Wang et al., 2012) or under ionic stress in *Psychroflexus torquis* ATCC 700755^T^ (Feng et al., 2013). It is worth to note that all these experiments were performed in batch cultures.

Combined, these previous results led Feng et al. (2013) to hypothesize that PR-based phototrophy might be important under stress conditions such as suboptimal pH or temperature, that divert energy from biosynthesis and growth to maintenance and survival. Currently maintenance energy can be estimated using chemostat experiments and Pirt’s model. This model is an extension of Monod’s (1950) classic chemostat model to which a maintenance term (maintenance coefficient, Pirt, 1965) is added. In practice the model (discussed in detail in the Supplementary materials) allows the measurement of maintenance to be calculated from a regression between the reciprocal of growth rates and the reciprocal of yields. Interestingly this model contrasts to the “textbook” notion in microbiology that microbial biomass in chemostats is independent of, or decreases with increases dilution rates, and just a product of the limiting substrate concentration and the yield on the limiting substrate.

Here we report on a study of where effects of light to PR-containing bacterium *Photobacterium angustum* S14 were tested using continuous culture conditions and carbon limitation. We hypothesized that whenever the continuous cultures were to be illuminated, some ATP would to be supplied to cells by PR-based phototrophy, leading to lower respiratory requirements and to more carbon available for biosynthesis. In this condition carbon yield should increase and the expectation would be that under illuminated conditions more biomass would be present in the culture vessels. Initially, we assumed that cultures followed the classic chemostat model and that yields were independent of dilution rates, but after results showed that this was not the case, we used Pirt’s theoretical framework to estimate maintenance levels and show that increases in carbon yields with light were only observed under conditions leading to higher maintenance energy levels.

## Materials and Methods

### Bacterial Strain and Growth Conditions

The PR-containing strain *P. angustum* S14 (formerly *Vibrio* sp. S14) was isolated in June 1981 from surface waters (1 m depth) of Botany Bay, NSW, Australia (Humphrey et al., 1983). Its genome has been sequenced (Lauro et al., 2009) and contains the entire PR-opsin and retinal biosynthetic pathway. This strain has been widely used as a model marine bacterium, as a copiotroph (Lauro et al., 2009; Williams et al., 2009) and as a model for UV resistance studies (Matallana-Surget et al., 2009).

Prior to all inoculations, the strain was pre-cultured in the dark at 25°C and 110 rpm in 50 ml of same medium as that used for continuous culture growth. Inoculation of the continuous cultures occurred at approximately after 8 h, when pre-cultures reached an optical density at 620 nm (OD_620 *nm*_) of about 0.7 (under “high” substrate conditions defined below) and 0.4 (under “low” substrate conditions defined below), measured with a model 1200 spectrophotometer (Fisher Bioblock, France).

### Continuous Culture Experiments

Changes in carbon yield *Y* (ratio of mg POC l^–1^ in the reaction vessels/mg C in glucose l^–1^ in the inflow medium) of *P. angustum* S14 were tested in duplicate at two substrate concentrations. “High” substrate medium was prepared in artificial seawater (ASW, Eguchi et al., 1996) using glucose (3 mM) as the sole carbon source, NH_4_Cl (9.35 mM) and NaH_2_PO_4_ (0.33 mM) as sources of N and P yielding a final molar C:N:P ratio of 54:28:1 to assure stoichiometric carbon limitation. For the “low” substrate medium, glucose, NH_4_Cl and NaH_2_PO_4_ were diluted fivefold. All continuous culture experiments were performed in Labfors Lux 3 photobioreactors (Infors HT, Switzerland). Inflow was controlled with an external peristaltic pump (Masterflex, USA) to provide fresh media. Outflow was controlled by the bioreactor’s peristaltic pump and by drawing the media at a height that kept the continuous culture at a constant 1 L volume. Temperature of the bioreactor was set to 25°C, aeration was provided by a vacuum pump/compressor (KNF Neuberger, Germany) with a rate of 250 ml per minute and was stirring at 300 rpm, leading to oxygen saturation above 90% in all vessels. Fluorescent bulbs (14 W Grolux, F8WT5-GRO, Sylvania, UK) were used as light sources giving a light intensity (Photosynthetic Active Radiation) of 406.7 μE · m^–2^ · s^–1^ measured in the media prior to inoculation with a model QSL irradiance meter (Biospherical Instrument Inc., USA).

Based on preliminary experiments that showed *P. angustum* S14 was inhibited by continuous illumination under certain media formulations (Courties, 2013), we measured carbon yields in duplicate vessels using 12 h light: 12 h dark cycles for each of the substrate levels [Vessels H1 and H2 (“high” substrate); Vessels L1 and L2 (“low” substrate)] and compared to yields in single controls ran in continuous darkness for each of the substrate levels [Control H (“high” substrate); Control L (“low” substrate)]. 10 ml of the pre-culture were transferred into the bioreactor and the strain was grown in batch mode to an OD_620 *nm*_ of about 0.4 (“high” substrate condition) and to an OD_620 *nm*_ of about 0.15 (“low” substrate condition) measured as described above, at which time the feed and outflow pumps were started. For all experiments the target dilution rate was of 0.1 l · h^–1^ corresponding to about a quarter of the maximum batch growth rate of *P. angustum* S14 using the “high” substrate medium. Oxygen saturation, temperature and pH were followed using built-in sensors of the bioreactor (Infors HT, Switzerland). Once OD_620 *nm*_ was stabilized (c.a. between 90 and 110 h), triplicate samples for particulate organic carbon (POC), particulate organic nitrogen (PON) and cell counts were collected (these samples were considered as equivalent to those in the dark control), and at this stage the light was turned on. For the following six 12 h light: 12 h dark cycles, triplicate samples for OD_620 *nm*_ and cell counts were taken about every 4 h and samples for POC every 12 h (near the end of the light or dark phases). Sampling was stopped for the following five cycles and sampling was restarted at the same regime from cycles 12 and 13. For the dark controls OD_620 *nm*_, cell counts and POC sampling were taken at times equivalent to those taken in the treatments. Actual dilution rates were estimated by measuring the volume of outflow integrated over 24 h.

### Batch Experiments

In order to obtain growth characteristics of *P. angustum* S14 in batch mode, and in conditions equivalent to those used in the continuous culture experiment, we performed duplicate growth curves in the light (406.7 μE · m^–2^ · s^–1^) and in the dark using the photobioreactors, the “high” substrate medium and the same parameters used in the continuous culture experiments. Growth was estimated from OD_620 *nm*_ measurements as above.

The optimum pH for growth was tested in duplicate using the “high” substrate medium to which buffers MES (Sigma-Aldrich; for pH 5.5–6.5), HEPES (Sigma-Aldrich; for pH 7.0–8.0), or AMPSO (Sigma-Aldrich; for pH 8.5–10.0) were added at 2.0 mg/ml. pH was adjusted to a 5.5–10.0 range using 25% (v/v) concentrated HCl or 1 N NaOH. Growth was followed in 24 well plates (Evergreen Scientific, USA) incubated at 25°C in the dark at 100 rpm. OD_620 *nm*_ measurements were obtained using a Paradigm™ Multi-mode Detection Platform (Molecular Devices, USA).

### POC and PON Measurements

Particulate organic carbon and PON were measured from triplicate samples using a standard protocol: a volume of 5 ml (“high” substrate experiments) and 10 ml (“low” substrate experiments) were filtered through glass fiber filters (GF/F, Whatman, UK), which were finally placed in glass vials, dried for 8 h, and acidified with concentrated HCl for at least 8 h and dried again. All used filters as well as glass vials had been pre-combusted at 450°C for at least 12 h. Carbon and nitrogen was measured with a Series II 2400 CHNS/O Analyzer (Perkin-Elmer, USA) using a modified Dumas method (Jacobs, 1965). Final POC and PON values were subject to an outlier analysis where any of the triplicate samples whose value was higher than twofold or lower than 0.5 of the standard deviation of the remaining two samples was considered as an outlier. These values were removed from the analysis.

### FACS and Optical Density Measurements

Triplicate 1 ml samples were fixed with 1% glutaraldehyde (Sigma-Aldrich, USA), flash frozen in liquid nitrogen and stored at –80°C. Samples were diluted 100-fold and stained for 15 min with a 1:400 dilution of SYBR green I (Molecular Probes, USA). Cell counts were measured in triplicate by flow cytometry using a FACSCalibur flow cytometer (Becton Dickinson, USA) equipped with an air-cooled argon laser (488 nm, 15 mW) as previously described (Van Wambeke et al., 2011).

**FIGURE 1:**
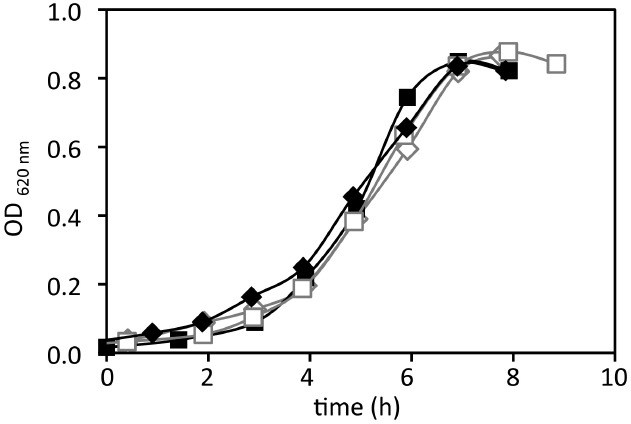
**Growth in batch of *P. angustum* S14 in photobioreactor batch experiments using “high” substrate conditions.** Duplicates growth in dark (black curves) and in the light (406.7 μE · m^–2^ · s^–1^, gray curves).

Final OD_620 *nm*_ measurements (shown in all figures, except for Figure 1) were taken in triplicate in 96 well microplates from the same fixed samples using a Paradigm™ Multi-mode Detection Platform (Molecular Devices).

### Glucose Measurements

Residual glucose in the medium was measured in triplicate (filtrate of the filters used for POC measurements) using a Glucose Assay kit (Sigma-Aldrich) adapted to a 48 well-microplate format with OD_540 *nm*_ measured using a Paradigm™ Multi-mode Platform (Molecular Devices). Since, as expected, all measurements from experimental Vessels H1 were in the micromolar range, thus negligible compared to the concentration of the inflow medium, a subset of samples was measured in triplicate (dark and light samples in cycle 13, and the last sample for the controls) for the remaining experiments.

### Data Analyses

Statistical analyses were performed using the R^1^ software. Estimation of values of the maintenance coefficient, as well as the modeling used to evaluate quasi steady state conditions was scripted and ran using scilab^2^. Details of these analyses are presented in the supplementary methods.

## Results

In order to evaluate the effect of light on growth of *P. angustum* S14 in batch and estimate growth parameters, batch experiments were run in photobioreactors with the same medium, substrate, agitation and aeration as those used in continuous culture experiments at “high” (i.e., 3 mM glucose) substrate levels. In these conditions *P. angustum* S14 grew at a rate of approximately 0.4 h^–1^ and the growth rates were not significantly different (ANCOVA, *p* = 0.71) between illuminated (0.442 ± 0.047 h^–1^) and dark (0.468 ± 0.047 h^–1^) conditions (Figure 1).

### Continuous Culture Experiments: “High” Substrate Levels

**FIGURE 2:**
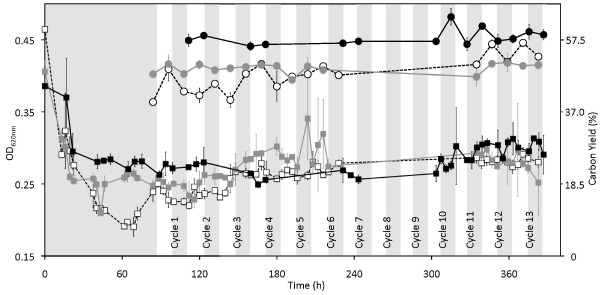
**Effect of cyclic 12 h illumination and 12 h darkness on the biomass and carbon yield of *P. angustum* S14 grown in chemostats under “high” substrate conditions.** Gray and white vertical bars represent dark and light periods respectively for treatment chemostats, onto which data from the dark-only control were plotted based on sampling time. White squares, gray squares and black squares represent OD_620 *nm*_ values for Vessel H1, Vessel H2, and Control H, respectively. Open circles with dotted lines, gray circles with gray lines and black circles with black lines represent carbon yield in percentage for Vessel H1, Vessel H2, and Control H, respectively.

**FIGURE 3:**
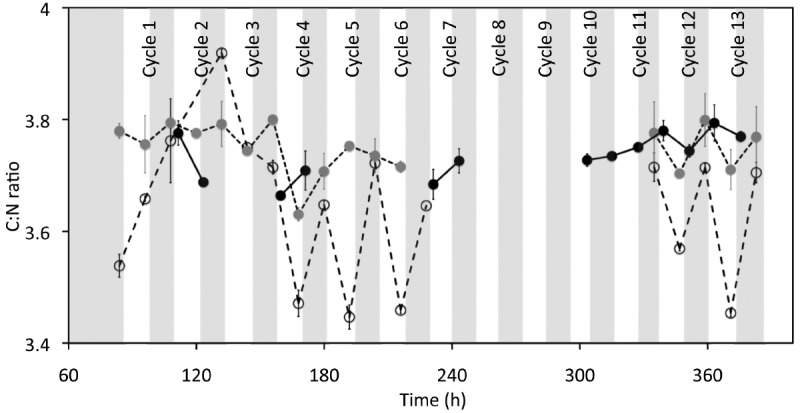
**Effect of cyclic 12 h illumination and 12 h darkness on the C:N ratio of *P. angustum* S14 cells grown in chemostats under “high” substrate conditions.** Vertical bars are as in Figure 2. White circles, gray circles and black circles represent C:N ratios for Vessel H1, Vessel H2, and Control H, respectively.

The first set of continuous cultures was run on what we called “high” substrate levels. The progression of biomass in two replicate continuous cultures (Vessel H1 and Vessel H2) under 12 h light and 12 h dark cycles, and in a control continuous culture kept in the dark (Control H), is shown in Figure 2. We observed that after about 80 h, the OD_620 *nm*_ and cell counts (Figure S1 in supplementary material) were relatively stable (within ± 0.5 × of the average values) except in Vessel H1 where OD_620 *nm*_ and cell counts increased with a corresponding decrease in pH at ca. 140 h, a time corresponding to a new batch of medium being used (Figure S1 in supplementary material; arrow). In all vessels the pH was low (ca. 5.0–6.0) when compared to the optimum pH for growth of *P. angustum* S14 (pH 8.0–9.0; data not shown) reflecting the relatively high cell counts (ca. 0.5–1.0 · 10^9^ cells · ml^–1^). Throughout 13 light–dark cycles all parameters were relatively stable in both replicates as well as in the control. We observed nonetheless a slight increase in POC, and thus carbon yield, throughout the entire experiment for Vessel H1, as well as some increases after 12 h illumination, particularly during cycles 12 and 13 in Vessel H1 and during early cycles in Vessel H2 (Figure 2). We also observed clear drops (ca. 0.1–0.2) in C:N ratios after 12 h of illumination in both illuminated vessels (Figure 3). In these conditions both POC and PON increased but relatively PON increased more, leading to a drop of the ratio (Figure S2 in supplementary material). These variations were not observed in the control vessel (Figure 3). Treating each light–dark cycle as a replicate measure, this decrease was very significant (ANOVA, *p* < 0.001) for Vessel H1 and significant (*p* = 0.022) for Vessel H2 after cycle 4. Glucose concentrations in all measured samples were in the micromolar range (about 1:1000 than that of the inflow media), and thus it was assumed to be 0 in subsequent calculations.

One remarkable observation was that the average POC values, and thus *Y* (expressed in % hereafter), were significantly higher (ANOVA, *p* < 0.001) in the dark control (56.1 ± 2.25), than either of the treatment Vessels [46.8 ± 0.003 (H1) and 48.2 ± 0.015 (H2)] and very importantly, this difference existed even when only comparing values prior to the start of light–dark cycles (Figure 2). Apart from the obvious difference in light regimes between the treatment vessels and the control, we observed that the dilution rates were not maintained perfectly constant during experiments (Figure S3 in supplementary material). Variations in dilution led the continuous culture to a sequence of quasi-steady states that allowed measurements under different dilutions.

**FIGURE 4:**
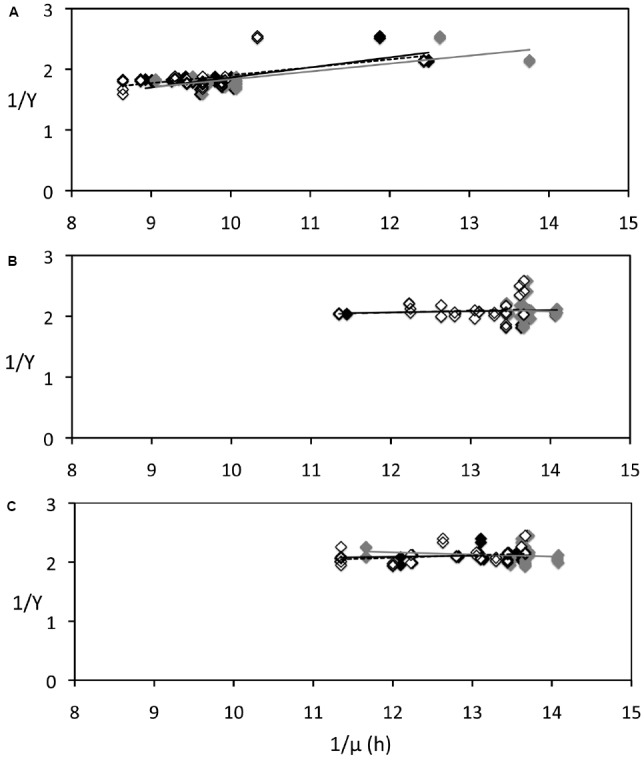
**Relationship between carbon yields (*Y*) and growth rate of *P. angustum* S14 grown in chemostats under “high” substrate conditions used to estimate Pirt’s maintenance coefficient.** Growth rates were estimated from integrated dilution rates measured prior (gray diamonds, gray regression lines) or after (open diamonds, dotted regression lines) *Y* samples were taken, or using interpolated values (black diamonds, black regression lines). **(A)** Cells kept in continuous darkness until sampling. **(B)** Cells sampled after 12 h of illumination (406 μE · m^–2^ · s^–1^). **(C)** Cells sampled after 12 h darkness.

We used Pirt’s theoretical framework (Supplementary Methods) and estimated *m* values from a regression line between 1/*Y* and 1/μ, assuming the system was at steady state (i.e., μ= *D*, the dilution rate). To evaluate the maintenance coefficient under different conditions, regressions parameters were calculated for *Y* values measured in continuous darkness (in both replicates and in the control; R_*DD*_), *Y* values measured after 12 h illumination (R_*LC*_), and *Y* values measured after 12 h darkness (R_*DC*_). Since *D* was measured at different times at a lower frequency than *Y*, three regressions lines were calculated using three different values of *D* [prior (R_*DD*–*P*_, R_*LC*–*P*_, R_*LC*–*P*_), after the *Y* measurement (R_*DD*–*A*_, R_*LC*–*A*_, R_*LC*–*A*_), and using interpolated *D* values (R_*DD*–*I*_, R_*LC*–*I*_, R_*LC*–*I*_)]. The results (Figure 4) show that *m* was higher (0.142 ± 0.020 h^–1^) when *Y* was measured in continuous darkness (Figure 4A) than both when *Y* was measured after 12 h illumination (*m* = 0.005 ± 0.036 h^–1^; Figure 4B) or after 12 h darkness (*m* = 0.007 ± 0.040 h^–1^; Figure 4C), suggesting that light–dark cycles might have led to a decrease of the maintenance coefficient of *P. angustum* S14.

**FIGURE 5:**
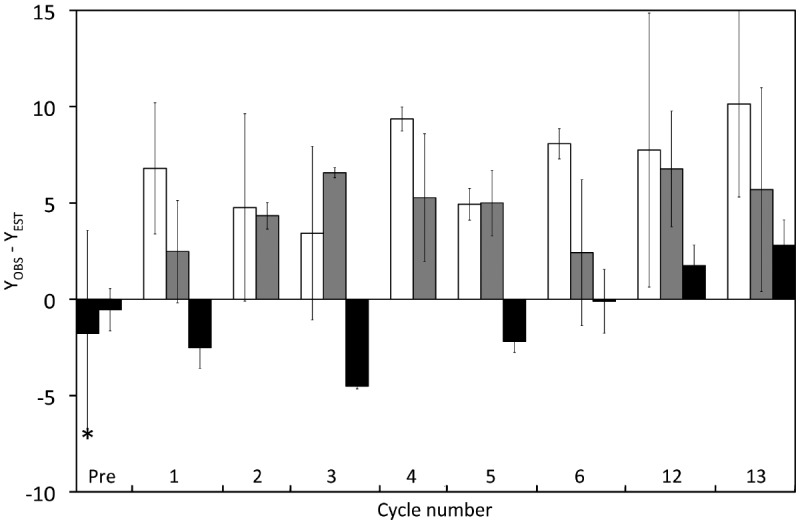
**Effect of cyclic 12 h illumination (406.7 μE · m^–2^ · s^–1^) and 12 h darkness on the carbon yield of *P. angustum* S14 grown in chemostats under “high” substrate conditions.** The *Y*_*OBS*_–*Y*_*EST*_ anomaly corresponds to *Y* values corrected for the effect on *Y* of varying dilution rates. Dark bars: Cells kept in continuous darkness until sampling. Light bars: Cells sampled after 12 h of illumination. Gray Bars: Cells sampled after 12 h darkness. The asterisk corresponds samples from Vessel H1 and Vessel H2, prior to the start of light–dark cycles.

Since we observed an effect of varying dilution on yields, we performed a correction of *Y* before testing if light–dark cycles led to differences in carbon yields. This correction removed the confounding effect of variations dilution rates. We estimated what we called expected yields (*Y*_*EST*_) from interpolated *D* values using regression R_*DD*–*I*_ (black diamonds in Figure 4A). These values were then subtracted from the measured *Y* values (*Y*_*OBS*_) yielding an anomaly (*Y*_*OBS*_–*Y*_*EST*_) that allowed a comparison of yields under light and dark conditions, independent of variations in *D* (Figure 5). It is clear that this anomaly was significantly lower for samples taken in continuous darkness (0.23 ± 3.71) than for samples taken after 12 h illumination or after 12 h darkness (ANOVA; *post hoc* Tukey’s test; *p* < 0.001). The anomaly was not significantly different between samples taken after 12 h of illumination (6.91 ± 4.21) and those taken after 12 h of darkness (5.05 ± 3.14) based on the ANOVA *post hoc* Tukey test (*p* = 0.10), but was significantly higher (*p* = 0.043) using a pairwise *t*-test.

### Continuous Culture Experiments: “Low” Substrate Levels

A second set of experiments was run at what we called “low” substrate levels (600 μM glucose, same C:N:P ratio). As for the “high” substrate levels experiments, two replicate continuous cultures under 12–12 cycles (Vessel L1 and Vessel L2) and a dark control continuous culture (Vessel L) were performed. Contrary to our expectation, we did not observe the same effects of light on carbon yields and stoichiometry of *P. angustum* S14 under a lower level of substrate addition. OD_620 *nm*_ values were more scattered compared to the “high” substrate, particularly for the illuminated bioreactors, but this was the case even before the light was turned on (Figure S4 in supplementary material). Throughout the cycles a very slight decrease in OD_620 *nm*_, also reflected in a decrease POC values was observed. C:N ratios showed a steady decrease after illumination arriving at a value of about 3.4 in illuminated bioreactors, but there were no clear light–dark variations such as those observed in the “high” substrate experiments (Figure S5 in supplementary material). Levels of pH were much higher (Figure S6 in supplementary material) than those in “high” substrate experiments (Figure S1 in supplementary material), very likely due to the lower cells numbers (Figure S6 in supplementary material). These lower cell numbers and higher pH might partly explain the significantly higher *Y* values in the “low” substrate experiments (mean of controls and treatments 62.01 ± 2.73) compared to “high” substrate experiments (mean of controls and treatments 49.96 ± 5.02; ANOVA, *p* < 0.001).

**FIGURE 6:**
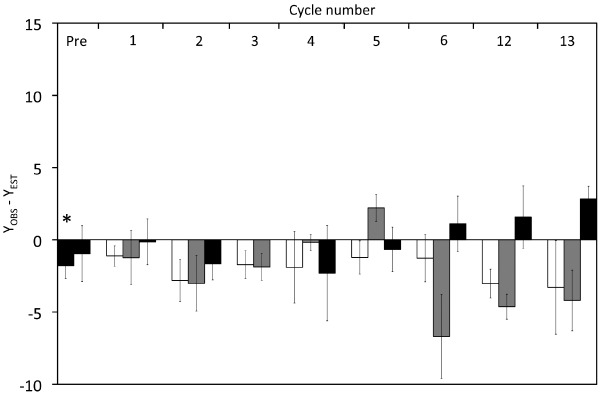
**Effect of cyclic 12 h illumination and 12 h darkness on the carbon yield of *P. angustum* S14 grown in chemostats under “low” substrate conditions.** The *Y*_*OBS*_–*Y*_*EST*_ anomaly corresponds to *Y* values corrected for the effects of dilution rates on *Y*. Back bars: Cells kept in continuous darkness until sampling. White bars: Cells sampled after 12 h of illumination. Gray Bars: Cells sampled after 12 h darkness. The asterisk corresponds samples from Vessel L1 and Vessel L2, prior to the start of light–dark cycles.

We calculated the maintenance coefficient in the same way as in the “high” substrate experiments (Figure S7 in supplementary material), and *m* values were low, as *Y* appears to be nearly independent of μ for the dark samples (*m* = 0.019 ± 0.007 h^–1^) as well as samples collected after 12 h illumination (*m* = 0.011 ± 0.001 h^–1^) or after 12 h darkness (*m* = –0.018 ± 0.008 h^–1^). For comparison sake we calculated the anomaly (*Y*_*OBS*_–*Y*_*EST*_) shown in Figure 6. Except for one value, all anomaly values from Vessels L1 and L2 were negative and *Y*_*OBS*_–*Y*_*EST*_ calculated from Vessels L1 and L2 were significantly different to those in Control L shown by an ANOVA (*p* < 0.001) and a pairwise *t*-test (*p* ≤ 0.001).

## Discussion

As previously shown for the other members of the family *Vibrionaceae* (Gomez-Consarnau et al., 2010; Wang et al., 2012; Akram et al., 2013), *P. angustum* S14 shows no responses to light under “normal” batch growth, and this is becoming the norm for these organisms, whereas the picture is more complicated for the *Flavobacteriaceae* even when the same genus is considered (Gomez-Consarnau et al., 2007; Riedel et al., 2013). The reasons for this discrepancy remain elusive and thus the examination of another strain with a sequenced genome such as *P. angustum* S14 will provide additional data to studies of comparative genomics targeting this question.

In addition, our initial working model assumed that *P. angustum* S14 cells would respond to substrate limitation in continuous cultures and modify their metabolism to use light as a supplementary energy source. This assumption did not appear to hold for this PR-containing prokaryote. This is in contrast to recent studies that measured carbon yields of the anoxygenic aerobic photoheterotroph *Erythrobacter* sp. NAP1 using continuous cultures (Hauruseu and Koblizek, 2012) which observed quite large differences in yield under illuminated conditions (dark and light cycles), and in agreement with previous theoretical predictions (Kirchman and Hanson, 2013). It appears that, at least for *P. angustum* S14, responses in *Y* to light are not solely dependent on carbon limitation. While it might be argued that the 3 mM of glucose is a high concentration relative to environmental conditions, high concentrations were needed to yield enough biomass (POC) that could be measured in a small enough volume that would not significantly interfere with the work volume of the continuous cultures. Furthermore, it is important to point out that residual glucose in the bioreactors were measured in micromolar concentrations, and that the C:N ratios were low, indicating that these cells were very likely carbon limited.

A number of responses to light (or to dark–light cycling) were observed in the “high” substrate bioreactor experiments that either did not occur, or were different to the “low” substrate bioreactor experiments. We observed significant increases of the *Y*_*OBS*_–*Y*_*EST*_ anomaly (ca. 6.0%) after light–dark cycles compared to full darkness, which were not observed in the “low” substrate experiments. This anomaly represents an increase in yield of about 15% relative to the *Y*_*EST*_ values calculated from dilution rates. However, since dilutions and yields were clearly higher in dark controls, and the slopes of the regression in Figure 4A were strongly influenced by pre-cycle values of Vessels H1 and H2, these values should be viewed conservatively. A second observed response only seen in “high” substrate was that in several cycles, we observed higher *Y*_*OBS*_–*Y*_*EST*_ after 12 h of illumination compared to the previous or subsequent 12 h in darkness, and the differences between *Y*_*OBS*_–*Y*_*EST*_ after 12 h illumination or after 12 h darkness were significant, albeit only for the *t*-test. Again, these differences were not observed in the “low” substrate experiment. Finally, very clear and significant decreases in C:N with illumination only seen in the “high” substrate experiments suggests a change in the physiology of *P. angustum* S14 with light, possibly leading to an increase in protein content relative to other cellular biomolecules with light.

Thus, it is clear that most of *P. angustum* S14 responses to light or light–dark cycles happened only under higher substrate levels that yielded higher biomass and led to lower pH levels. While we cannot unequivocally attribute these responses to pH or any specific factor, both the lower *Y* values in “high” substrate bioreactor experiments, as well as estimates of *m* from dark controls and pre-cycle samples, point to a higher cost of maintenance in these bioreactors compared to those in the “low” substrate additions, and in agreement with the suggestions by Feng et al. (2013) based on an analysis of several previous studies, that PR (thus light) can have an effect in conditions leading to “diversion of energy from growth and biosynthesis to maintenance and survival functions.” Since the original range of dilution rates used to calculate *m* was narrow, we performed additional experiments (“high” substrate, dark only) to measure *m* at a wider range of dilution rates. Interesting, while calculated *m* values were similar for *D* in the 0.075–0.1 h^–1^ range, lower *D* values (0.05–0.02 h^–1^) yielded lower biomass and higher (>6.0) pH levels. Values of *m* were clearly lower for those samples further supporting the effects of pH on maintenance.

Finally photoinhibition such as that observed in *V. campbellii* BAA-1116 (Wang et al., 2012) could be suggested as an alternative explanation to the observed lower values of yields in illuminated continuous cultures compared to those in the control, but this seems unlikely the case since (1) lower POC (thus *Y*) were observed previous to the first cycle, (2) POC (thus *Y*) increased or stayed relatively stable after 13 cycles, while a decrease would be expected under photoinhibition, (3) the anomaly *Y*_*OBS*_–*Y*_*EST*_ was always higher in illuminated continuous cultures when compared to the control, and (4) inhibition of growth rates would have led to relatively higher residual substrate at steady state than we observed.

We did not directly measure the expression of the PR-coding gene or of genes involved in the retinal biosynthetic pathway and thus we cannot assign these effects to PR proton pumping, but this remains as one of the most parsimonious explanations. The light levels used in our experiments were high compared to most of the previous experiments (Giovannoni et al., 2005; Gomez-Consarnau et al., 2007, 2010; Kimura et al., 2011; Wang et al., 2012; Akram et al., 2013; Feng et al., 2013; Riedel et al., 2013), and were chosen based on preliminary experiments (albeit with a different media and continuous light) showing that lower light levels (224 μmol of photons · m^–2^ · s^–1^) did not elicit responses that were observed at 406.7 μE · m^–2^ · s^–1^. Assuming that PR activity mediated the observed light responses by *P. angustum* S14, since this bacterium was originally isolated from surface waters, responses under these high light levels is not unexpected. Additionally, the PR of *P. angustum* S14 is predicted to be mainly a blue-absorbing PR based on the presence of a glutamine at spectral tuning position 105 (EBAC31A08 numbering). Blue-absorbing PRs retrieved from the Ocean surface were shown to have faster photocycles, indicative of adaptation to higher irradiances (Sabehi et al., 2005), even though this is not true for blue-absorbing PRs retrieved from deeper in the water column (e.g., Beja et al., 2001; Wang et al., 2003).

If confirmed in other PR-containing prokaryotes, the relationship between the maintenance coefficient and light responses by *P. angustum* S14 could be used to evaluate whether light is actually playing a role in PR-containing prokaryote metabolism in the environment, based on measurements of the maintenance coefficient of naturally occurring bacterioplankton under different light conditions. To date, few studies have attempted to estimate maintenance costs of mixed bacterioplankton assemblages (Cajal-Medrano and Maske, 1999, 2005). In these studies measurements were made for entire communities, using much lower dilution rates and levels of carbon addition, complicating comparisons to our data. In the latter study (Cajal-Medrano and Maske, 2005) measured *m* values (0.33 h^–1^) were higher than those we measured for *P. angustum* S14 and assuming these values are the same for PR-containing prokaryotes and other microorganisms, light-driven metabolism could contribute to fulfill maintenance requirements of PR-containing prokaryotes in the environment. The combination of molecular identification using probes and possible single cell estimates of respiration in dark versus light conditions, if developed, could provide better estimates of the contribution of light specifically to the metabolism of PR-containing prokaryotes in the future.

Finally our results suggest that unlike *Dokdonia* sp. MED134 (Gomez-Consarnau et al., 2007) responses to light by *P. angustum* S14 do not appear to be inversely correlated to substrate levels or limitation, but rather to the overall energetic balance of the cells, and thus again assuming these responses are caused by PR activity, biochemical mechanisms sensing energy levels rather than substrate levels might be mostly involved in the regulation of production and/or activity of PR-opsin together with its cofactor retinal. Future transcriptomic or proteomic studies with *P. angustum* S14 could lead to a better understanding of this regulation.

## Author Contributions

MS, AC, and PL designed the experiment. AC performed or significantly participated in all of the experimental work. TR performed most glucose analysis as well as OD measurements and POC analysis. AC, AR, and MS were responsible of continuous culture modeling and statistical analysis. AC, TR, MS, AR, and PL wrote the manuscript, approved its final version and agree to be accountable for all aspects of the work.

### Conflict of Interest Statement

The authors declare that the research was conducted in the absence of any commercial or financial relationships that could be construed as a potential conflict of interest.

## Footnotes

1 http://www.r-project.org

2 https://www.scilab.org/

## Abbreviations

PR: proteorhodopsin
*Y*_*OBS*_: measured values of carbon yield
*Y*_*EST*_: carbon yield estimated from dilution rate, assuming a correlation between *Y* and *D*.
