# Supplementary material for: Light-driven increase in carbon yield is linked to maintenance in the proteorhodopsin-containing Photobacterium angustum S14
Source: Front Microbiol. 2015 Jul 10;6:688. doi: 10.3389/fmicb.2015.00688 (PMC4498439; doi:10.3389/fmicb.2015.00688)
Supplement: Supplementary file 1 [file Presentation1.PDF]

Supplementary Material

Light-driven increase in C yield is linked to maintenance in the proteorhodopsin-containing Photobacterium angustum S14

Alicia Courties[1], Thomas Riedel[2†], Alain Rapaport[3.4], Philippe Lebaron[2], Marcelino T. Suzuki[2*]

[1] Sorbonne Universités, UPMC Univ Paris 06, CNRS, Laboratoire d'Océanographie Microbienne (LOMIC), Observatoire Océanologique, F-66650, Banyuls/mer, France
[2] Sorbonne Universités, UPMC Univ Paris 06, CNRS, Laboratoire de Biodiversité et Biotechnologie Marines (LBBM), Observatoire Océanologique, F-66650 Banyuls/Mer, France
[2] INRA-Supagro UMR MISTEA, Montpellier, France
[4] INRA-INRIA MODEMIC team, Sophia-Antipolis, France

***Correspondence**: Prof. Marcelino T. Suzuki, (Associate Editor, Frontiers in Microbiology)
Sorbonne Universités UPMC Univ. Paris 6 and CNRS, UMR 3579, LBBM, Observatoire Océanologique, Avenue du Fontaulé, Banyuls-sur-Mer, 66650, France
suzuki@obs-banyuls.fr

[†] Current address: Department of Microbial Ecology and Diversity Research, Leibniz-Institute DSMZ – German Collection of Microorganisms and Cell Cultures, Braunschweig, Germany

1. Supplementary Methods

Chemostat modeling and numerical simulation.

The classical chemostat model (1) describes the time evolution of a population of microorganisms and of a single limiting resource in continuous culture or in the so-called "chemostat" experimental device:

$$\begin{cases} \dfrac{dx}{dt} = \mu(s)x - Dx \\ \dfrac{ds}{dt} = -\dfrac{\mu(s)x}{Y} + D(s_{in} - s) \end{cases}$$

where x and s describe respectively the concentrations of biomass and of the limiting substrate. D is the dilution rate and s_{in} the input concentration of substrate. The strain is characterized by its specific growth rate $\mu(.)$ and the yield coefficient Y. Monod has proposed to use the Michaelis-Menten growth function for $\mu(.)$:

$$\mu(s) = \frac{\mu_{max} s}{K_s + s}$$

where μ_{max} is the maximal growth rate and K_s the affinity constant. The mathematical analysis of this model predicts that for a dilution rate D less than $\mu(s_{in})$, the system reaches asymptotically a steady-state $(x*, s*)$ given by the following equations:

$$\mu(s^*) = D$$
$$x^* = Y(s_{in} - s^*)$$

(For dilution rates D larger than $\mu(s_{in})$, the system goes to the wash-out of the biomass). One can immediately deduce from these equations that $s*$ is increasing with D, which implies that x^* has to decrease with D.

Pirt (2) proposed to extend the Monod model adding a maintenance term mx that is subtracted from the substrate compartment:

$$\begin{cases} \dfrac{dx}{dt} = \mu(s)x - Dx \\ \dfrac{ds}{dt} = -\dfrac{\mu(s)x}{Y} + D(s_{in} - s) - mx \end{cases}$$

where m is a maintenance parameter. One can notice that steady states $(x*, s*)$ are still defined by the equation $\mu(s^*) = D$ for the substrate concentration at steady state, but now x^* is given by a different equation:

$$x^* = Y \frac{s_{in} - s^*}{1 + m\dfrac{Y}{D}}$$

As already noticed by Pirt, x^* is no longer a monotonic function of D:

- for small values of D, one has $x^* \cong D\frac{s_{in}}{Y}$, which is increasing with respect to D,
- for large values of D (provided that D less than $\mu(s_{in})$), one has $x^* \cong Y(s_{in} - s^*)$, as in the Monod model, that is x^* is decreasing with respect to D.

The ability of this model to describe a biomass at steady state x^* that increases with the dilution rate has led test the adequacy of the model to our dataset by using the variations in dilution rate and measured POC. We have first assumed that s^* is negligible compared to s_{in} (assumption that has was validated with all measurements of s), which implies that one can write:

$$\frac{x^*}{s_{in}} \cong \frac{m}{D} + \frac{1}{Y}$$

from which parameters m and $\frac{1}{Y}$ have been estimated with a linear regression between the inverse of the carbon yield $\frac{s_{in}}{x(t_i)}$ and the inverse of the dilution rate $D(t_i)$, observed at various times t_i. Notice that one can write equivalently:

$$D\frac{x^*}{s_{in}} \approx \frac{D}{Y} + m$$

which amounts to have a linear regression between the consumption $D(t_i)\frac{x(t_i)}{s_{in}}$ and the dilution rate $D(t_i)$ at various times t_i. In practice, these two regressions are not equivalent in the determination of the parameters m and Y, because of noise and imperfection, and we have chosen the one that provided the smallest residue of the least square adjustment.

Nevertheless, some precautions have been necessary to justify the consideration of the model at steady state:

- In the chemostat experiments, the time varying feature of the dilution rate did not fulfill the hypothesis of the classical chemostat, for which D is constant trough the experiment. Consequently, we have checked that these variations were sufficiently slow to validate a quasi steady state approximation of the model (this amounts to consider that at each time t_i of measurement of the biomass, the system is really about a steady state).
- The measurement of the time varying dilution rate was not available on line, but between successive times $\tilde{t}_{j-1}, \tilde{t}_j$, the exact volume $\Delta V(\tilde{t}_{j-1}, \tilde{t}_j)$ extracted from the chemostat has been measured, providing an estimation of the dilution rate at time $\tilde{t}_j$:

$$D_{estim}(\tilde{t}_j) = \frac{\Delta V(\tilde{t}_{j-1}, \tilde{t}_j)}{\tilde{t}_j - \tilde{t}_{j-1}}$$

  Notice that the times $\tilde{t}_j$ were different from the measurement times t_i. So one has also to estimate D at times t_i.

The assumption of quasi-state has been checked through numerical simulations: provided a time history of the dilution rate $D(.)$, we have checked on the simulations of the model that $\mu(s(t_i)) \cong D(t_i)$ and $x(t_i) \cong x^*(t_i)$ at each measurement time t_i. Prior knowledge of the function $\mu(.)$ and of the parameters of the model was thus necessary:

- the parameters μ_{max} and K_s of the function $\mu(.)$ were estimated from experiments in batch culture,
- the value of s_{in} was known,
- the parameter Y and m have been estimated under the steady state and the linear regressions described previously,
- the initial condition $\left(x(0), s(0)\right)$ was known.

We have also checked that the choice of estimators of the dilution rate for any time (that is necessary for running the model simulations) do not impact significantly the results. For any time t, we first find the index j such that $\tilde{t}_{j-1} \leq t < \tilde{t}_j$, and consider the three possible estimations:

- $D_{estim}(t) = D(\tilde{t}_{j-1}) + \frac{D(\tilde{t}_j)-D(\tilde{t}_{j-1})}{\tilde{t}_j-\tilde{t}_{j-1}} (t - \tilde{t}_{j-1})$

or

- $D_{estim}(t) = min\left(D(\tilde{t}_{j-1}), D(\tilde{t}_j)\right)$

or

- $D_{estim}(t) = max\left(D(\tilde{t}_{j-1}), D(\tilde{t}_j)\right)$

References

1. Monod J (1950) La technique de culture continue: Théorie et applications. *Ann. Inst. Pasteur* 79:390-410.

2. Pirt SJ (1965) The maintenance energy of bacteria in growing cultures. Proc R Soc Lond B Biol Sci 163(991):224-231.

1. Supplementary Figures

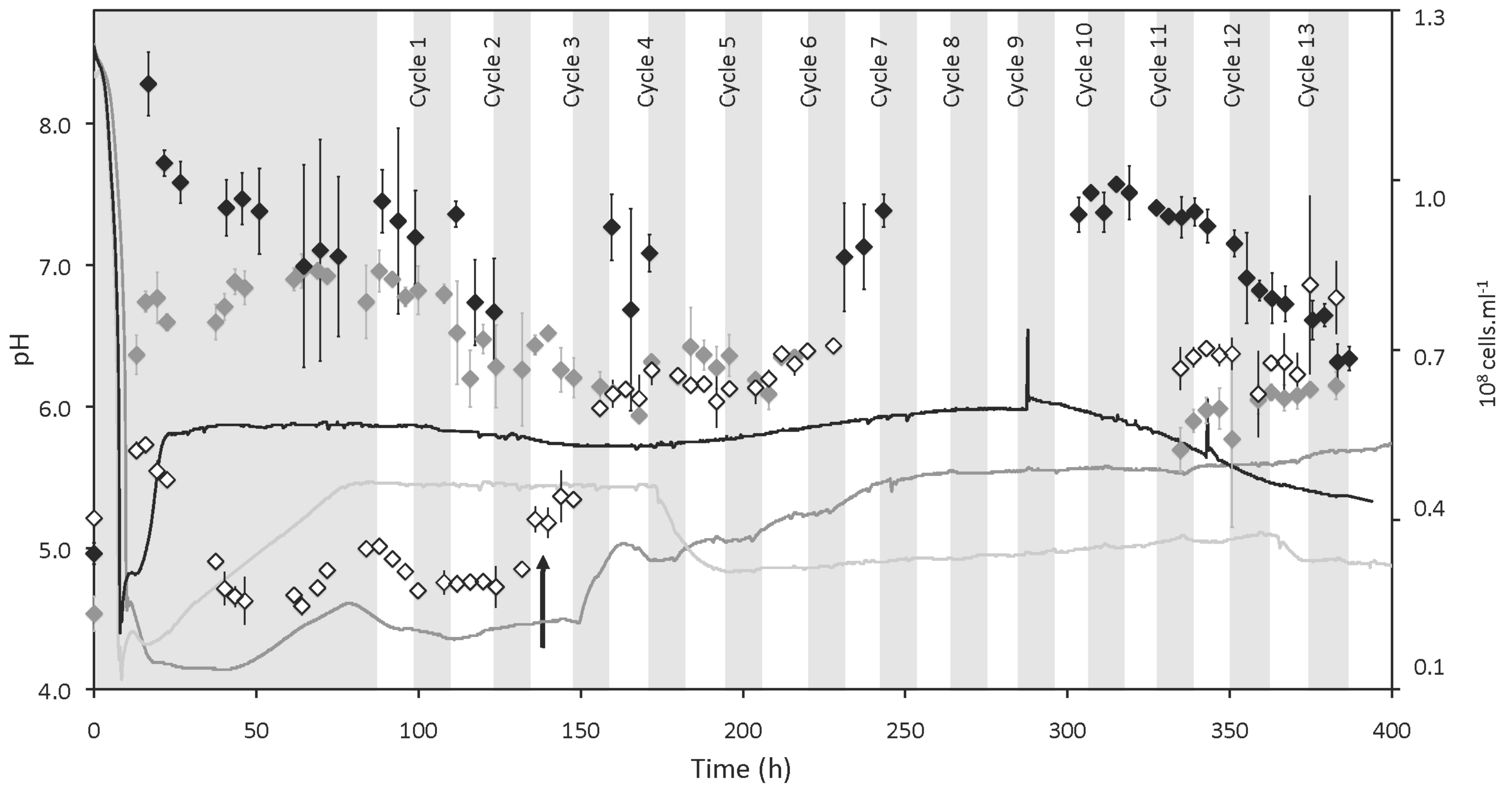

Figure S1. Effect of cyclic 12 h illumination and 12 h darkness on the cell numbers of *P. angustum* S14, and vessel pH in chemostats under "high" substrate conditions. Grey and white vertical bars represent dark and light periods, respectively for treatment chemostats, onto which data from the dark-only control were plotted based on sampling times. White diamonds, grey diamonds and black diamonds represent cell counts values for Vessel H1, Vessel H2 and Control H respectively. Light grey, dark grey and black lines represent pH values for Vessel H1, Vessel H2 and Control H, respectively. The arrow indicates a time when a new batch of medium was used.

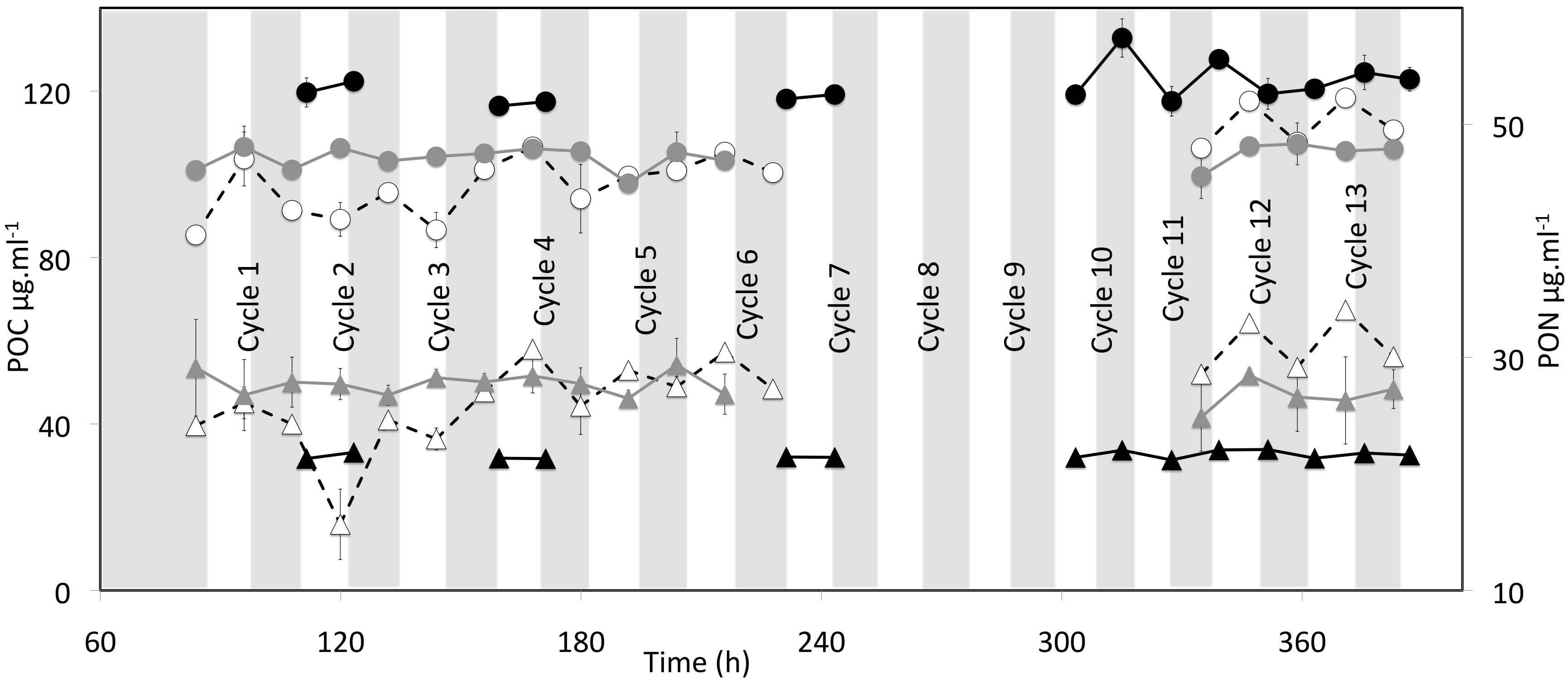

Figure S2. Effect of cyclic 12 h illumination and 12 h darkness on POC and PON in *P. angustum* S14 cultures grown in chemostats under "high" substrate conditions. Vertical bars are as in Figure S1. White circles, grey circles and black circes represent POC values for Vessel H1, Vessel H2 and Control H, respectively. White triangles, grey triangles and black triangles represent PON values for Vessel H1, Vessel H2 and Control H, respectively.

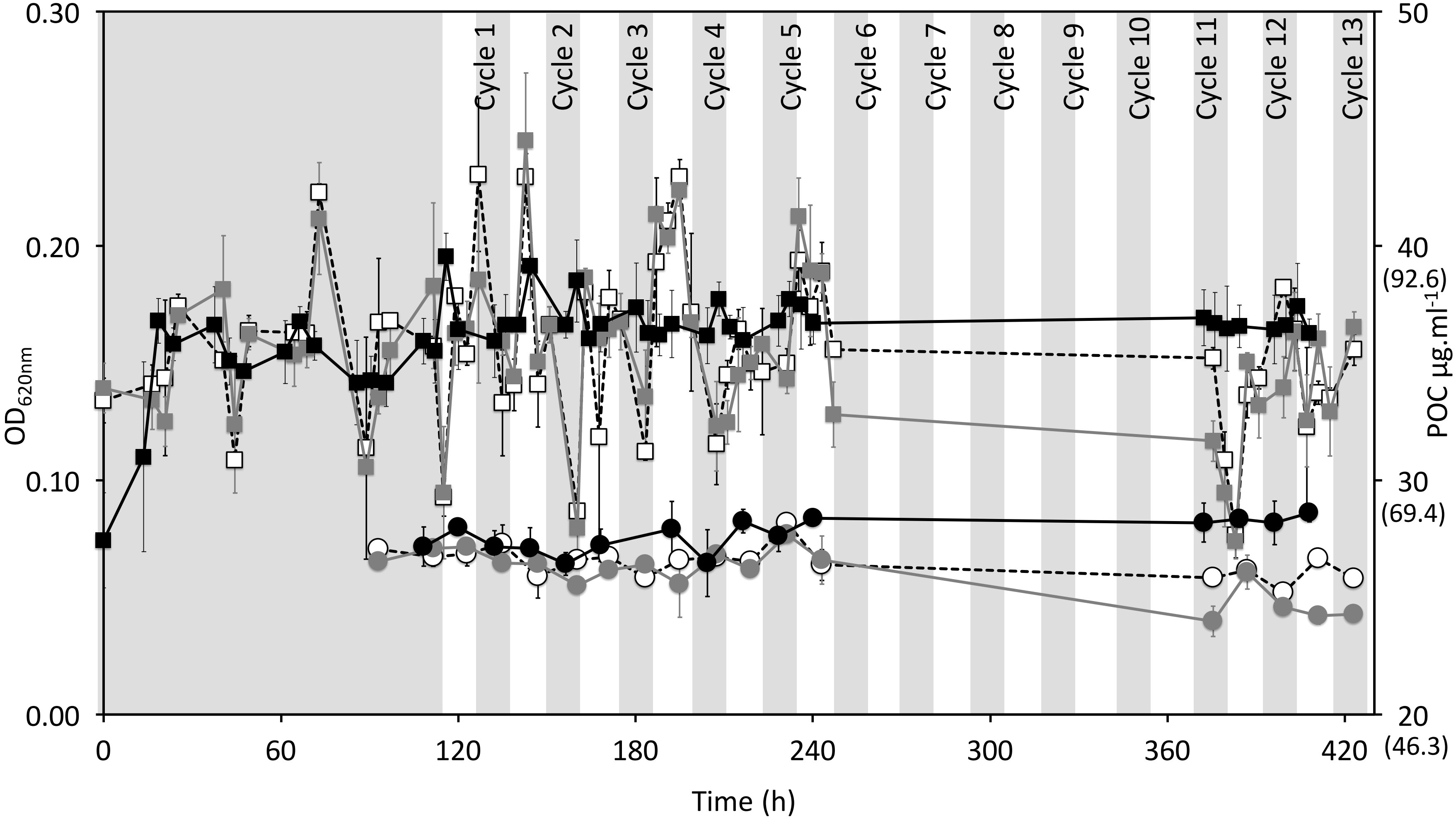

Figure S4. Effect of cyclic 12 h illumination and 12 h darkness on the biomass of *P. angustum* S14 grown in chemosta "low" substrate conditions. Vertical bars are as in Figure S1. White squares, grey squares and black squares represent (values for Vessel L1, Vessel L2 and Control L respectively. White circles, grey circles and black circles represent POC for Vessel L1, Vessel L2 and Control L, respectively and with equivalent percentageYield values in parenthesis.

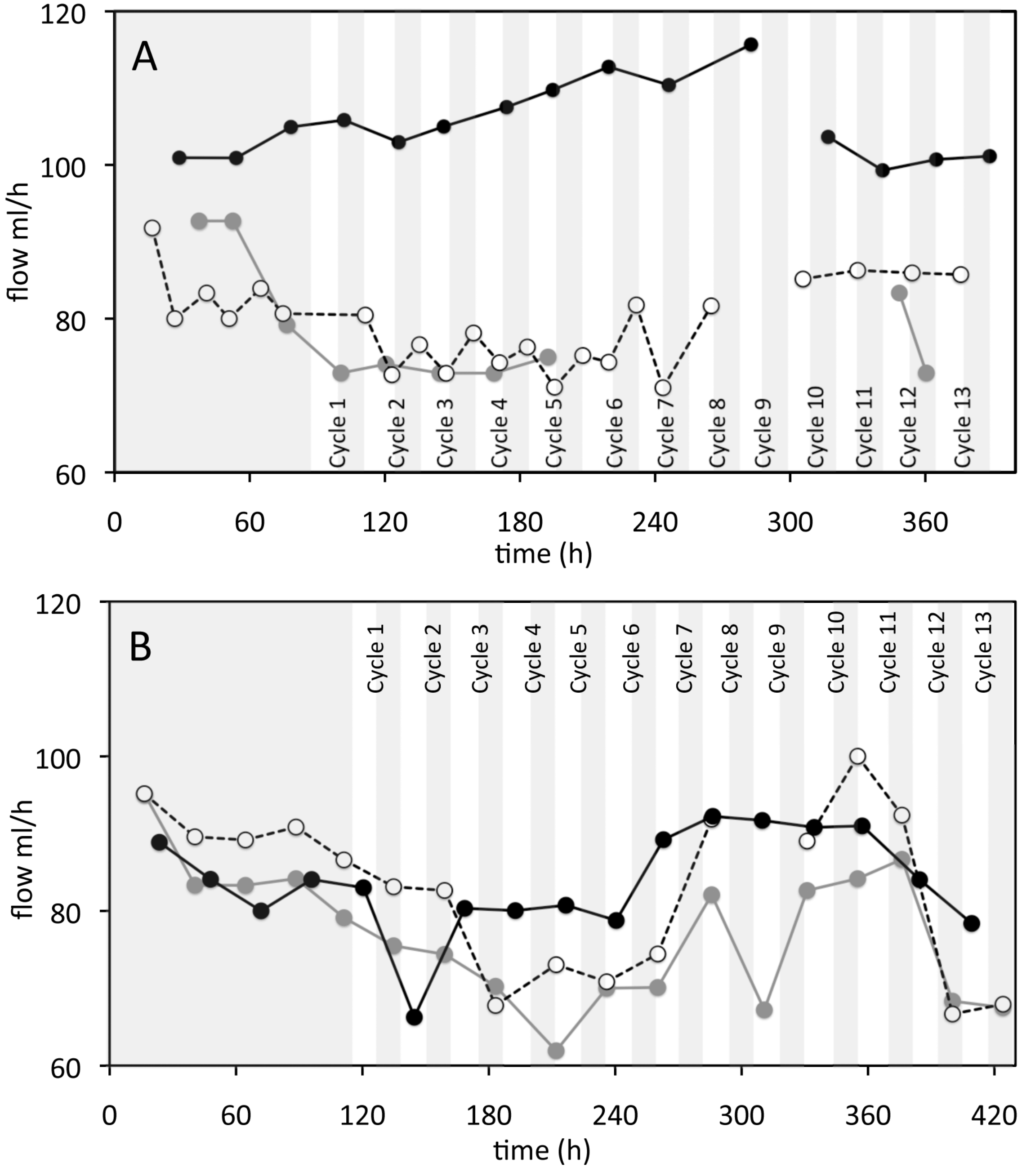

Figure S3. Measured flow rates of chemostats. Vertical bars are as in Figure S1. A) “high” substrate conditions. B) “low” substrate conditions.

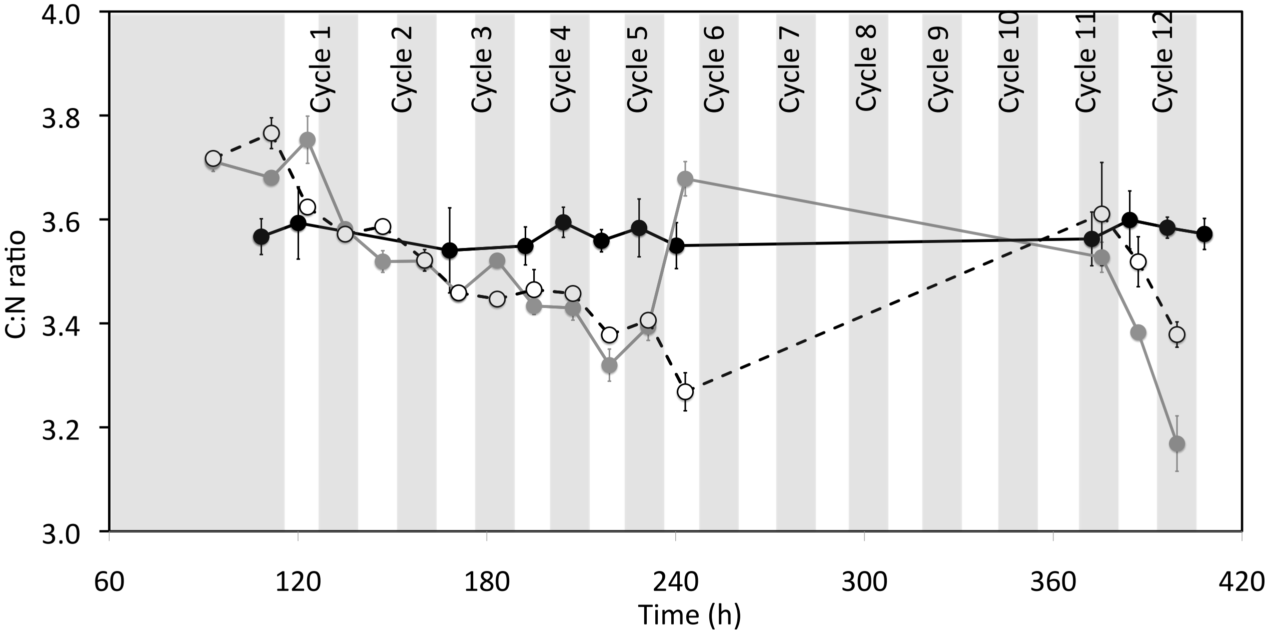

Figure S5. Effect of cyclic 12 h illumination and 12 h darkness on the C:N ratio of *P. angustum* cells S14 grown in chemostats under "low" substrate conditions. Vertical bars are as in Figure S1. White circles, grey circles and black circles represent C:N ratios for Vessel L1, Vessel L2 and Control L, respectively.

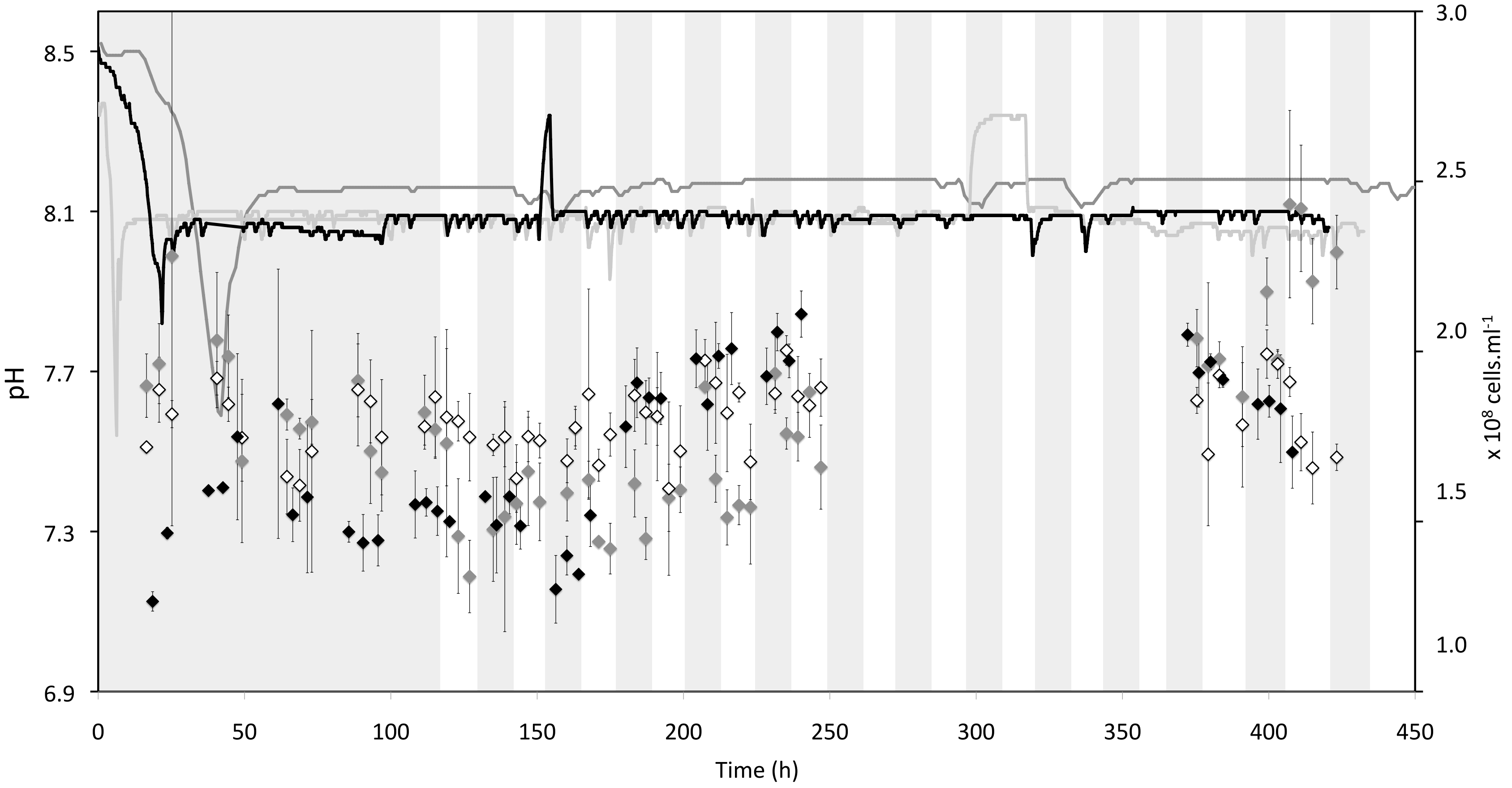

Figure S6. Effect of cyclic 12 h illumination and 12 h darkness on the cell numbers of *P. angustum* S14 and vessel pH in chemostats with "low" substrate conditions. Vertical bars are as in Figure S1. White diamonds, grey diamonds and black diamonds represent cell counts values for Vessel L1, Vessel L2 and Control L, respectively. Light grey, dark grey and black lines represent pH values for Vessel L1, Vessel L2 and Control L, respectively.

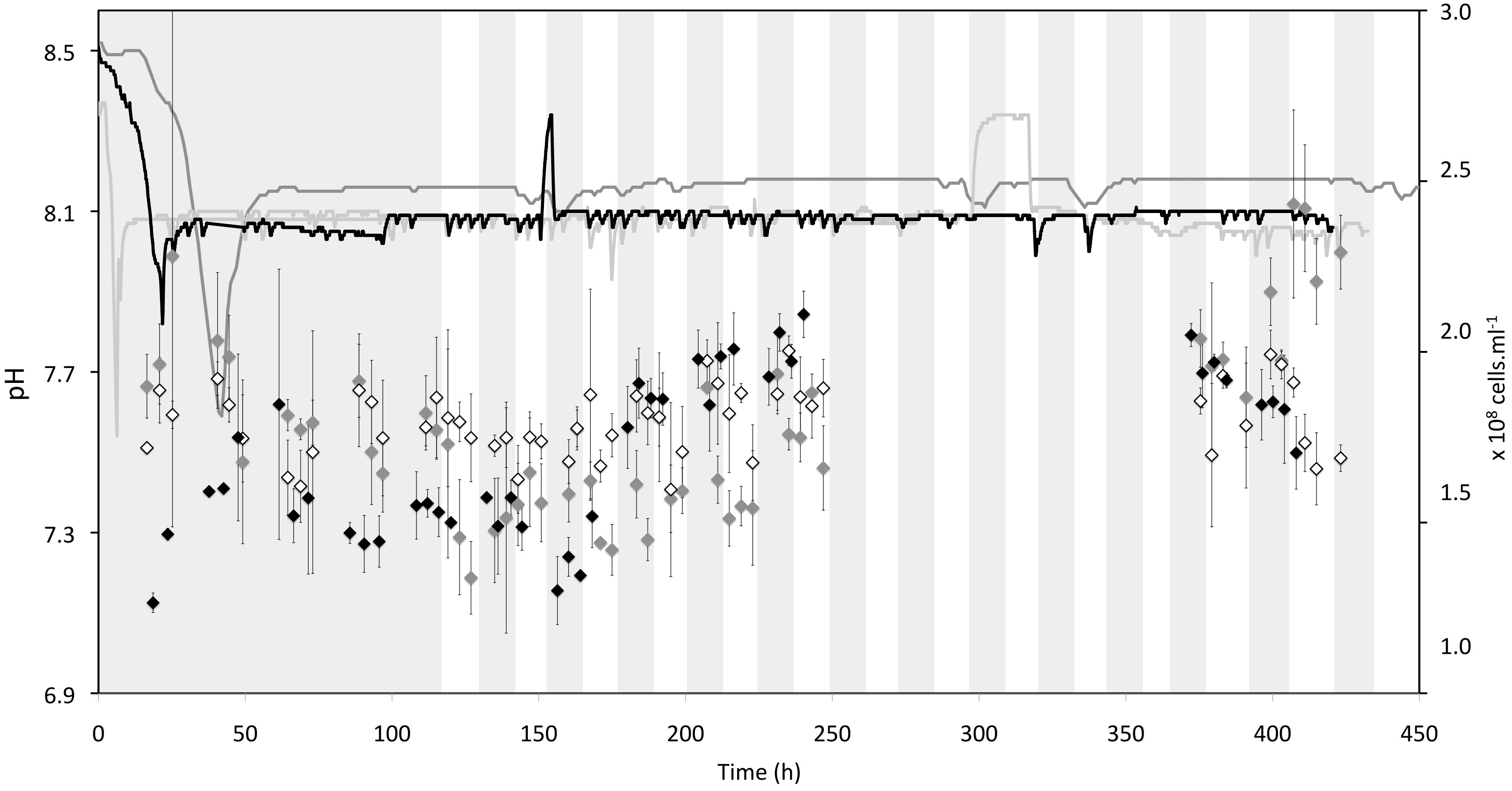

Figure S7. Effect of cyclic 12 h illumination and 12 h darkness on the cell numbers of *P. angustum* S14 and vessel pH in chemostats with “low” substrate conditions. Vertical bars are as in Figure S1. White diamonds, grey diamonds and black diamonds represent cell counts values for Vessel L1, Vessel L2 and Control L respectively. Light grey, dark grey and black lines represent pH values for Vessel L1, Vessel L2 and Control L, respectively.
